# Schwertmannite: occurrence, properties, synthesis and application in environmental remediation

**DOI:** 10.1039/c8ra06025h

**Published:** 2018-10-01

**Authors:** Zhuo Zhang, Xue Bi, Xintong Li, Qiancheng Zhao, Honghan Chen

**Affiliations:** Beijing Key Laboratory of Water Resources & Environmental Engineering, School of Water Resources and Environment, China University of Geosciences Beijing 100083 China; Beijing Junmei Environmental Technology Co., Ltd. Room 1505. Tower B, New Logo International Tower, No. 18A, Zhongguancun South Street, Handian District Beijing, 100081 China bixue@bjjunmei.com.

## Abstract

Schwertmannite is a typical iron-derived mineral, which was originally discovered in acid mine drainings and subsequently synthesized in the laboratory. Increasingly, it is seen as having considerable potential as an adsorbent material, which could be used for environmental remediation (such as the treatment/remediation of arsenic, chromium, antimony, fluoride, and organic contaminants). This study reviews current developments, mainly in the preparation, structure, and water treatment of Schwertmannite. Several key issues are discussed in detail, such as synthetic strategy, the structure–property relationships, potential environmental applications, and related mechanisms. Soil remediation by schwertmannite is compared to water treatment, and its application is further evaluated. Finally, the methodologies for water treatment and soil remediation using schwertmannite are also taken into consideration from an environmental point of view.

## Abstract 2

Schwertmannite is a typical iron-derived mineral, which was originally discovered in acid mine drainings and subsequently synthesized in the laboratory.

## Graphical abstract

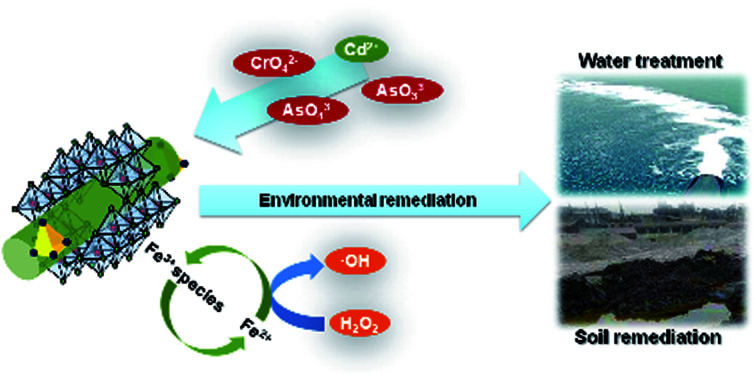

## 1. Introduction

Schwertmannite (Sch) is a secondary iron (Fe)-hydroxylsulfate mineral, which is found in acidic sulfate-rich environments. It could potentially be used to treat multiple heavy metals in contaminated water or soils. It is probably one of the most frequently observed minerals that precipitate from ferriferous aqueous systems, such as mine drainages, soil solutions, and lake water,^1^ similar to ferrihydrite.

### 1.1 Discovery

In natural environments, the Fe–sulfur (S) system automatically cycles through a reduction path based on Fe sulfide and an oxidation path based on Fe sulfate. The Fe sulfates are commonly found in natural marine environments or lakes, *etc.*, which often also contain Sch.^2^ Natural sources of Sch can be acid mine drainings (AMD), acid sulfate soils (ASS),^3^ solid minerals, such as ochreous sediments,^4^ natural Fe and S oxidation by-products in coal,^5^ and some types of industrial liquid/solid waste produced during human activities, such as copper heap leachate solutions.^6^

Early studies on Sch were undertaken by Brady *et al.*^7^ and Murad^8^ before it was recognized as a discrete mineral. There was no chemical analysis. Therefore, Sch was tentatively identified as a “well” crystallized ferrihydrite phase. In 1990, Bigham *et al.*^9^ discovered a poorly crystallized oxyhydroxy-sulfate of Fe in sediment samples collected from mine effluents in the eastern Ohio coalfield and drainage exiting an abandoned copper–arsenic mine in Finland, which was identified as the primary component of ochreous precipitates from sulfate-rich mine waters in the pH range of 2.5–4.0. This material was approved as an independent mineral species by the Commission on New Materials and Mineral Names of the International Mineralogical Association in 1992 (90-006).^10^ Two years later, Bigham *et al.*^10^ reported its occurrence at the Pyhäsalmi sulfide mine, Province of Oulu, Finland. In the same year, this mineral was named in honour of Udo Schwertmann, Professor of Soil Science from the Technical University of Munich, who published the first description of Sch.^9^ In 1995, after being frequently identified in precipitates from acid mine waters, a fully natural Sch was found in a small stream draining a pyritic Schist at PfitScher Joch, Zillertaler Alps, Austria.^11^ In Japan, Sch was discovered in 2003, based on a phenomenon of naturally attenuated arsenic (As) concentration form abandoned As mine in Nishinomaki,^12^ and then the first patent about Sch for absorbent was published.^13^ In Chinese, Sch is called “shī weī tè màn shí”,^14^ and was first found in AMD sediments from the Malan coal mine, Shanxi Province, China, in 2006,^15^ and afterwards at the Dabaoshan mine in South China.^16–19^ The research development of Sch and the primary contributions of researchers or groups are listed in Table 1. The table shows that natural Sch was discovered, identified, and structurally classified within the first few years of its discovery. After this, related theoretical studies (over nearly 20 years) about its properties and further application, especially for environmental protection, began to attract more attention.

**Table 1:** Research development into schwertmannite over 25 years

Starting years	Authors	Groups	Contribution	Ref.
1990	J. M. Bigham	J. M. Bigham	Discovered Sch in acid mine draining and studied on the Sch's structure	9
1994	J. M. Bigham	E. Murad	The occurrence in Pyhäsalmi sulfide mine, Province of Oulu, Finland.	10
1994	IMA&CNMMN	—	Official approval	10
1995	U. Schwertmann	J. M. Bigham	A fully occurrence in a stream draining of Austria	11
1996	G. A. Waychunas	G. A. Waychunas	AsO_4_^3−^ and SeO_4_^3−^ substituted Sch	49
2003	F. Keisuke	F. Keisuke	The discovery of Sch in As mine	12
2004	Oishi	—	The first patent about Sch for absorbent	13
2004	S. Regenspurg	S. Regenspurg	Chemical oxidation for Sch's preparation	20
2005	S. Regenspury	S. Regenspurg	AsO_4_^3−^ and CrO_4_^2−^ incorporation in Sch	29
2006	H. F. Sun	F. H. Zhao	Found in Shanxi Province, China	15
2006	P. Acero	P. Acero	Behavior of trace elements during Sch precipitation	81
2009	E. D. Burton	E. D. Burton	Sorption of As(v) and As(iii) by Sch	32
2011	S. Paikaray	S. Paikaray	Removal of As(iii) using Sch upon various synthesis	38
2013	E. D. Burton	E. D. Burton	Microbially mediated reductive transformation of Sch for As removal	65
2013	W. M. Wang	X. Han	Sch as a new Fenton-like catalyst for oxidation of phenol	62
2014	C. L. Vithana	C. L. Vithana	Effect of fulvic acid during the arsenic removal by Sch	78
2015	F. W. Liu	W. H. Fan	Ferrous ion chemical oxidation: different H_2_O_2_ supply rates	31
2015	M. Q. Chen	G. N. Lu & Z. Dang	Sch's role during the natural sulfate migration	16

### 1.2 Chemical composition

The groups led by Bigham^9,10^ and Schwertmann^11^ proved experimentally that this type of mineral can be expressed as Fe_8_O_8_(OH)_8−2*x*_(SO_4_)_*x*_ (1 ≤ *x* ≤ 1.75), with a molar ratio for Fe/S of between 4.6–8.0, and with a mass percentage for SO_4_^2−^ of between 12.5%–20.5% in the theoretical chemical formula when crystal water is not considered. However, differences between operators, methods, and conditions mean that the mass percentage for SO_4_^2−^ can range widely from 5.3% (ref. 4) to 32%.^20^

The Fe species was proven to be entirely Fe^3+^ by the Murad group and others after using Mössbauer spectroscopy.^9,21–24^ This stable Fe chemical state gives this material a unique structure and could potentially be used in a number of different fields.

### 1.3 Microstructure

**Fig. 1:**
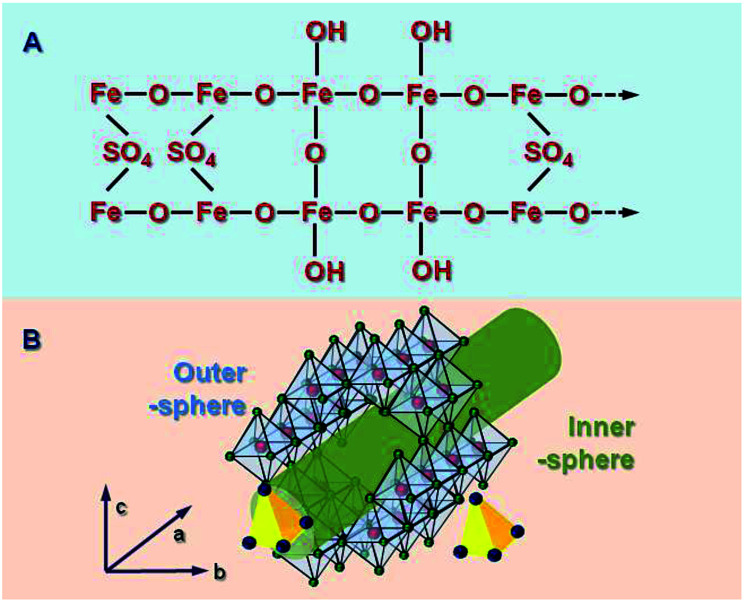
Chemical (A) and channel (B) structure of schwertmannite.

Characterization by X-ray diffraction (XRD) has suggested that Sch has poorer crystallinity characteristics than jarosite and goethite, but it is quite similar to ferrihydrite.^25^ However, Loan *et al.*^26^ demonstrated that most Sch whiskers have a structure that is consistent with maghemite, which is similar to some ferrihydrite components, whereas some sections also contain highly disordered ferrihydrite structural components and more amorphous regions. This description is mainly based on XRD data and some other direct images about the surface morphology of Sch. The Sch particles are transformed into crystal cubic and hexagonal phases, during which, sulfate and OH/H_2_O are ejected from the structure and sulfate is structurally incorporated. The microstructure of Sch is shown in Fig. 1A.

Schwertmannite is a novel mineral and possesses a 3D structure with ordered channels. Fukushi *et al.*^27^ studied previous reports and gave a comprehensive description of possible coordination modes, which involved a bidentate bridge of SO_4_^2−^ within an akaganéite-like structure. Sch has dominant crystal planes at {100},^9^ which feature double sequences of surface O atoms that are arranged in six rows, are coordinated with Fe, and oriented parallel to *c*, as in the following: Fe_3_O, Fe_1_O, Fe_3_OH, Fe_3_O, Fe_3_OH, and Fe_2_O. Four surface O atoms and –OH groups are present in the 1^st^, 2^nd^, and 5^th^ rows. In the 3^rd^ and 5^th^ rows, SO_4_ structures replace one –OH group to form a bridging bidentate (Fe_3_)SO_4_ surface group. Fernandez-Martinez *et al.*^28^ showed a deformed frame of an Fe octahedral that was similar to that of akaganéite. This was based on pair distribution functional data, XRD analysis, and density functional theory calculations. The simulations suggested that two sulfate molecules per unit cell were present in the structure, which formed one outer sphere and one inner sphere complex inside the channels created by the Fe octahedral.

The material is described as “analogous in structure to basic Fe sulfate, but with a –Fe–O– cage, as shown in Fig. 1B. The Sch possessed a channel-like structure in the *c* axis orientation with a diameter of *ca.* 0.50 nm.^5^ The SO_4_^2−^ ions in the channel pores can be easily substituted by other ions that have a similar radius, such as PO_4_^3−^ (*r* = 0.238 nm), CrO_4_^2−^ (Cr: chromium) (0.240 nm), or AsO_4_^3−^ (0.248 nm).^29^ Bigham *et al.*^30^ reported that SO_4_^2−^ ions have a mass percentage of between 10–15%. A third of them are adsorbed on the mineral surface and two-thirds are found in the channels.

## 2. Synthetic strategy – structural characteristics

### 2.1 Synthetic strategies

The most common formation process for natural Sch (formula (1)) usually goes through two main steps, which are the oxidation of Fe^2+^ and co-precipitation of Fe^3+^. However, after nearly 20 years of development and improvement, Sch can be synthesized using various techniques that involve the chemical oxidation of ferrous species, abiotic precipitation of ferrous species (fast hydrolysis and slow dialysis), and/or biological ferrooxidation of ferrous species by Fe^2+^-oxidizing bacteria, such as *Acidithiobacillus ferrooxidans*, as Table 2 listed. Essentially, Sch is an Fe mineral salt, which means there can be various Fe chemical sources, such as Fe^2+^ salts and Fe^3+^ salts, but only if the SO_4_^2−^ concentration is high enough. Usually, if the Fe source materials have a low valence, another oxidation step is necessary, whereas, when they are in a high valence state, then only phase transformation is needed.

1 8Fe(SO_4_) + 2O_2_ + (4 + *x*)H_2_O → Fe_8_O_8_(OH)_*x*_(SO_4_)*y*↓ + (8 − *y*)SO_4_^2−^ + 2(8 − *y*)H^+^

**Table 2:** The characteristics comparison of synthetic strategies for Sch samples

Synthetic methods	Fe^2+^ oxidation		Fe^3+^ hydrolysis	
	Chemical oxidation	Biological ferrooxidation	Fast hydrolysis	Slow dialysis
Fe source	Fe^2+^ salts (sulfate)		Fe^3+^ salts (sulfate, nitrate, chloride)	
Temperature	24–75	25–40	60–85	60
pH value	2.4–3.0	2.5–3.2	1.66–4.0	—
Oxidant	H_2_O_2_	—	—	—
Time	24 h	5–7 days	1 h–25 days	30 d
References	20,32,34,35,38,43,78,89	24,38,40,41,44,51,53,83,84,90	1,6,52,61,62,89,92	20,26,35,38,45,46,53,102,103

#### Chemical methods

Chemical methods for Sch preparation refer to the chemical oxidation of Fe^2+^ species and the abiotic precipitation of Fe^3+^ species, but these techniques are limited to basic theoretical research and small-scale synthesis in the laboratory. In these two methods Fe^3+^ undergoes precipitation, but the former one needs another prior oxidation step for Fe^2+^ species.

#### Chemical oxidation of Fe^2+^ species

This method involves two main steps: pH adjustment of the Fe^2+^ solution and the addition of H_2_O_2_. It was first published by Regenspurg *et al.*^20^ who followed a technique provided by Pentinghaus. In most associations, this method is carried out at room temperature. Previously, H_2_O_2_ has been the most used oxidant for the Fe^2+^-oxidation process due to its clean reaction (formula (2)). The addition rate for this oxidant into FeSO_4_·7H_2_O aqueous solution influences the formation of Sch, and a slow H_2_O_2_ supply significantly inhibits the total Fe precipitation efficiency, but improves the *S*_BET_ or AsO_3_^3−^ removal capacity of Sch.^31^ However, Sch synthesized by chemical oxidation with H_2_O_2_ usually appears as very fine particles with relatively low specific surface area (SSA) values that are within the range of 4–14 m^2^ g^−1^.^20,32^ Generally speaking, they can be nearly 50 m^2^ g^−1^ (ref. 33) or larger than 100 m^2^ g^−1^ (ref. 34) due to the effects of some aggregates with large diameters. The Sch samples obtained by this method may have much smaller and finer particles with quite weak XRD signals.^35^ The separation of Sch products are dependent on washing and fast centrifugation or filtration, the efficiency of which may be affected by the particle sizes.

2 2Fe^2+^ + H_2_O_2_ + 2H^+^ → 2Fe^3+^ + 2H_2_O

#### Abiotic precipitation of Fe^3+^ species

The Fe^3+^ cations in water can be precipitated both over a short period of time and constantly over the long-term. During the hydrolysis of Fe^3+^ species in infinity dilutions, the constants of the hydroxo complexes of Fe^3+^ were remarkably different when they were measured by Stefansson.^36^ In the precipitation process, the Fe^3+^ species firstly combines with water molecules to form complex intermediates, and then transforms into Fe-based oxysulfate (it becomes Sch when high concentrations of SO_4_^2−^ exist). This process is shown by formula (3).

3 Fe^3+^ + 6H_2_O → [Fe(H_2_O)_6_]^3+^8[Fe(H_2_O)_6_]^3+^ + (24 − 2*x*)OH^−^ + *x*SO_4_^2−^ → Sch + 56H_2_O

In comparison to the chemical oxidation method, the abiotic precipitation of Fe^3+^ species is carried out at higher temperatures, which are usually higher than 60 °C and lead to fast precipitation. Furthermore, the nucleation rate for abiotic precipitation is relatively higher than for chemical oxidation. Different abiotic precipitation techniques may lead to differences in crystal-growing time, but not the nucleation rate (fast completed in short time).

#### Fast hydrolysis

The Fe^3+^ solutions were subjected to homogeneous hydrolysis at pH ≥ 1 by Reichelt *et al.*^37^ As the compounds age, the, [Fe(H_2_O)_6_]^3+^ or [Fe(OH)_2_(H_2_O)_6_]^+^ complexes transform into hydroxo complexes, and then polymerize into low molecular and positively-charged polynuclear complexes, such as [Fe_2_(OH)_3_]^3+^ or [Fe_3_(OH)_2_]^7+^ before precipitation. The creation of fast precipitation processes (involving a complex hydration–decomposition–polymerization–aging–precipitation process) causes the formation of kinetically favourable morphologies with low crystallization levels. When the precipitate remains in suspension, a thermodynamic optimum is approached due to crystallization and structural changes. This technique has been used on a small-scale, but scaled-up equipment has already been produced by a group led by Bertau.^37^ They investigated the precipitation reaction of Fe_2_(SO_4_)_3_ solution with ammonia using a microjet mixer with gas discharge and a two impinging jet mixer to obtain nano-structured particles in the μm range. The fast hydrolysis method only requires a relatively fast wash and separation period for the final product, which may make it easier to create a working system, but could also bring problems, such as a disordered structure and inaccurate composition due to fast nucleation and incomplete washing. Previous studies have suggested that this strategy provides Sch products with similar particle sizes and XRD peaks to the ones obtained through chemical oxidation. This is probably due to the similar separation process for Sch.

#### Slow dialysis

In contrast to fast hydrolysis, slow dialysis has a remarkably long treatment time (usually longer than a month) for the crystal growing and product washing stages, which results in a longer reaction or crystallization time. The slow dialysis techniques for Sch synthesis involve long-term dialysis by cellulose membranes over about 30 days followed by a freeze drying process, which is expensive, complex, and time-consuming.^38^ However, the mineral particle diameters of the synthetic Sch produced by slow dialysis are larger than the synthetic Sch produced by the rapid methods. They also have better XRD signals and Fourier transform-infrared spectroscopy (FT-IR) characteristics.^35^

It needs to clear that all the three methods we mentioned above involves chemical phase-transformation of Fe^3+^, without interferes of other composites. Such processes need precise control of condition factors to make sure the purity and structure of aimed product. Yet, the process droved by biological force may more effective and eco-environmental.

#### Biological ferrooxidation

Exactly speaking, biological ferrooxidation is also an oxidation path which uses bacteria or microorganisms as mediates, different from the pure chemical path. Biogenic fabrication takes place at room temperature and is dependent on bacteria or microorganisms that use a common Fe^2+^ source as a feedstock. In this process, a high SO_4_^2−^ concentration drastically inhibits akaganéite bioformation, but facilitates the occurrence of biogenic Sch.^39^*Acidithiobacillus ferrooxidant* cells have traditionally been used in the past, but novel bacteria, such as an acidophilic Fe-oxidizing strain C25,^40^ can be used after it has been cultured or isolated from its original environment. Furthermore, this method can also be combined with acidic and alkaline activation,^41^ which may result in a higher SSA value, more hydroxyl groups, and inner-sphere sulfate complexes. Therefore, the biosynthesis pathway for Sch (bio-Sch) may be more effective when connected to other pathways. For example, bio-Sch combined with *Brevibacterium* sp. YZ-1 (used for microbial oxidation) has already been used to simultaneously treat As-contaminated soil,^42^ and is considered a green remediation strategy for remediating As-contaminated soils.

In summary, the chemical oxidation methods were the earliest Sch production methods, whereas slow dialysis and the biogenic method can be more efficiently upscaled. However, chemical oxidation is more suitable for theoretical studies, especially when investigating crystal structure and surface morphology, because of its stricter requirement for heterogeneous synthesis and separation of the product.

In nature, the final properties of Sch are not only dependent on the fabrication method, but are also closely tied with the post-processing procedure, which includes the separation, drying, and smashing processes. Usually, drying methods in the laboratory involve natural drying, heat drying, freeze drying, and air drying. Freeze drying is more suitable for stable repeated preparation when the diffusion and transfer of water molecules in the samples is taken into consideration. After the drying step, physical steps, such as smashing and grinding, are used to obtain the powdered sample. These processes change the surface colour. After the post-processing steps and the separation process mentioned above, a whole preparation procedure that uses slow fabrication and freeze drying may result in Sch products with better crystallinity and larger particle sizes.

### 2.2 Characteristics

Previously reported studies on Sch materials have mainly focused on the structure–properties relationship. However, it is necessary to discuss the structure-related reports on synthetic methods because the different structures/morphologies are closely dependent on the synthetic strategies used and their tunable parameters.

#### Morphology

Schwertmannite has a multiple three-dimensional structure that could be seen as a hierarchical model. Its particles range in size from the nanoscale to the microscale. The entire morphology can also vary. Previous reports suggested that there are mainly two morphologies. These are spheroids with a smooth surface, and hedge-hog-like particle and pin-cushion-like or sea urchin-like with a rough surface. Generally, a fast formation process results in a kinetically favoured structure containing spherical particles, whereas a slow fabrication process results in a thermally favoured structure with a hedge-hog-like morphology. Sometimes, secondary aggregates with large particle sizes are produced.

**Fig. 2:**
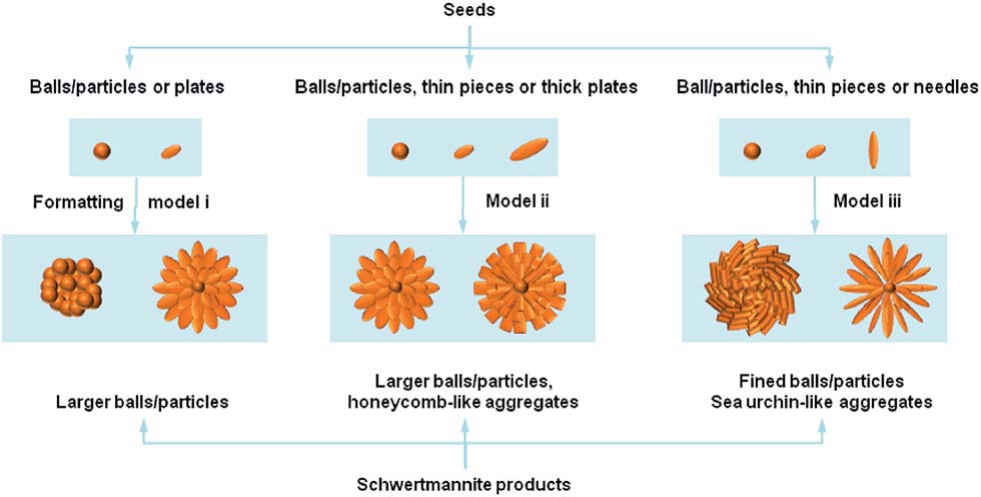
Schematic illustration of the formation process from seeds to schwertmannite products.

Fig. 2 shows that most of the Sch samples obtained by chemical oxidation contain spherical particles that are larger than 0.5 μm and at the μm scale. Models ii and iii produce particles that have a hedge-hog-like or a sea urchin-like morphology, which is possibly caused by a higher synthetic temperature or a slow addition rate for H_2_O_2_.

The reaction temperature is important in the oxidation process. French *et al.*^43^ stated that the synthetic Sch produced *via* chemical oxidation should not be described as a single phase with a repeating unit cell, but as a polyphasic nano-mineral with crystalline areas spanning less than a few nano-meters in diameter within a characteristic “pin-cushion”-like amorphous matrix. The difference in synthetic temperature affects the density of the needles on the Sch surface. The needles on Sch produced at higher temperatures had a dendritic morphology, whereas the needles on Sch produced at room temperature were more closely packed.

The H_2_O_2_ addition rate is intensively optimized for stable mineralization. Liu *et al.*^31^ reported that a slow H_2_O_2_ supply rate led to a low ferrous ions oxidation efficiency and low total Fe precipitation efficiency in Sch synthesis systems, which increased the SSA value by changing the morphology from a spherical to a “hedge-hog” structure. This improved the heavy metal ion removal efficiency.

There are too many examples of fast hydrolysis for an in-depth discussion. Model ii in Fig. 2 shows that two kinds of remarkably different morphologies can be produced. The first has a hedge-hog like structure with a diameter of *ca.* 100 nm, whereas the second type aggregates with each other and produces particles that cover a board range of sizes. After studying a large numbers of literatures, it is discovered that, the hedge-like morphology of Sch minerals are mainly formed at higher temperature (*ca.* 85 °C) without control of pH value, whereas the aggregated one of Sch minerals are mainly formed at lower temperature (<60 °C) and lower pH values (2.4–3.0). Considering the pH value are mainly decided by the detail strategies, such as lower pH values for oxidation reaction while relatively higher pH values for hydrolysis, it was firstly settled as an environmental factor. Different from that, the temperature factor may influence the nucleation and aging process of crystallization, which in great degree decides the final morphology of Sch products. Thus, once the pH condition has been set, the temperature factor becomes the main cause of morphology formation.

Another method involving slow dialysis is also based on the hydrolysis of Fe^3+^ species and is quite similar to fast hydrolysis. However, the precipitation time for the dialysis is much longer than the fast hydrolysis pathway. This process mainly utilizes an extra cellulose membrane, which requires consistent treatment of the precipitate. Fig. 2 shows that the samples produced by model ii all possess a typical hedge-hog like structure, which may be due to the use of 60 °C as the synthetic temperature. A higher temperature promotes dialysis, which thermally stabilizes the 3D structure.

In contrast, the biological ferrooxidation method can be easily classified and evaluated. This micro-organism mediated process is also an oxidation method. As model iii in Fig. 2 shows, this method produces many more defined particles than the other methods. However, these biogenic Sch particles have a limited SSA value of <50 m^2^ g^−1^, which may be due to the ultrafine Sch particles.^44^

#### Crystal structure

Schwertmannite is an amorphous mineral material, which means that its crystal structure is not as regular as other materials. This review has used classical characterizations by techniques such as XRD, IR, and extended X-ray-absorption fine structure (EXAFS) to provide a detailed description of the crystal structure.

#### X-ray diffraction spectroscopy

Previously, it was believed that Sch had a crystal structure that was similar to akaganéite (nominally β-FeOOH).^9^ This structure was then used as a model for structure simulation studies.^27^ However, some researchers believed that Sch particles (balls or whiskers) had a similar crystallinity to ferrihydrite or maghemite, although it also had some amorphous regions.^25,26^ In reality, most researchers believe that Sch is a low-crystallinity mineral.

**Fig. 3:**
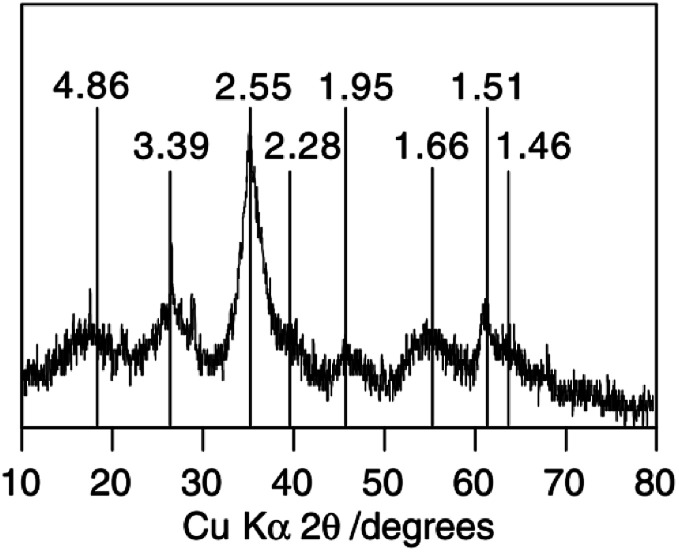
The classic XRD pattern of schwertmannite. (Reproduced from ref. 45 with permissions from the Elsevier).

The XRD data shows that Sch consists of 8-line diffraction profiles of between 0.486 and 0.146 nm (mainly at *ca.* 26.3, 35.2, 55.3, and 61.3°) as Fig. 3 shows.^45^ In all cases, the broad but consistent character of the diffraction maxima indicates a material that is poorly crystallized, but certainly not X-ray amorphous. However, the XRD diffraction patterns of the Sch samples are slightly different depending on the synthetic pathway used.

#### Infrared spectroscopy

Infrared spectroscopy is an important characterization technique and the species that have an infrared absorption ability are mainly Fe–O bands, O–H groups, and SO_4_^2−^ ions. The SO_4_^2−^ ions exist in more forms than Fe–O bands and O–H groups. The characteristic deformations and stretches of the O–H groups in Sch are at 860 cm^−1^ and 3300 cm^−1^, respectively.

**Fig. 4:**
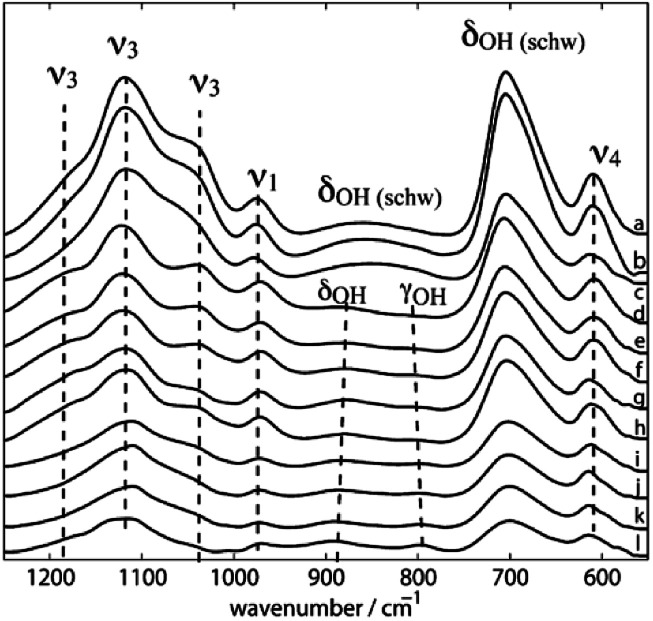
ATR FT-IR spectra of synthetic schwertmannite at different pH values at 298.2 K (a–l: 3.14, 3.44, 4.00, 4.40, 5.05, 5.50, 6.13, 6.52, 6.65, 8.04, 8.44, and 9.04). (Reproduced from ref. 46 with permissions from the American Chemical Society).

Three dominant SO_4_^2−^ groups are present in synthetic Sch according to the FI-IR spectra (Fig. 4) produced by Boily *et al.*^46^ which are tentatively assigned to H-bonded (I and III) and Fe-bonded (I) sulfate ions that are attached to Sch; and protonated (II) sulfate species with the following features: (I) bands at 1120, 1070, 1033, and 990 cm^−1^, which is similar to sulfate complexes adsorbed to hematite, goethite, and Sch in the presence of water; (II) bands at 1210, 1180, 1130, 1033, 972, and 966 cm^−1^, which is comparable to the sulfate spectra for hematite and has the greatest degree of *ν*_3_ splitting; and (III) bands at 1108 and 972 cm^−1^.

#### Extended X-ray absorption fine structure

**Fig. 5:**
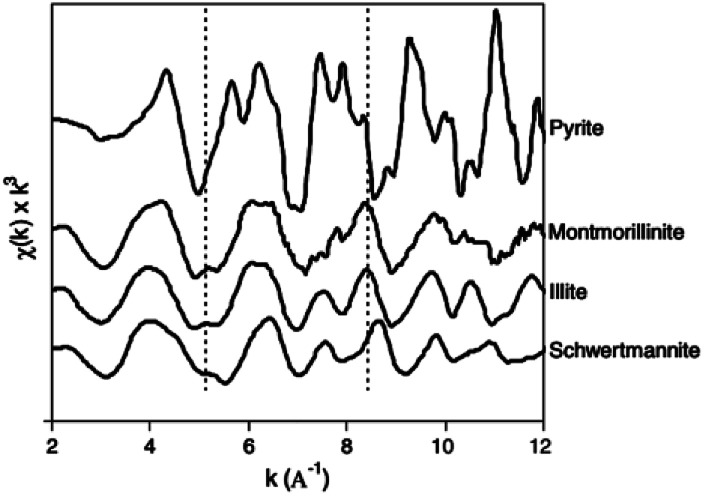
Iron K-edge EXAFS spectra of schwertmannite and reference minerals. (Reproduced from ref. 47 with permissions from the Elsevier).

The EXAFS technique is mainly used for Sch phase identification in natural sediments. Collins *et al.*^47^ combined analytical and field measurements to probe the speciation and cycling of Fe in coastal lowland ASS. Fe K-edge EXAFS (Fig. 5) demonstrated that Sch dominated (43–77%) 2^nd^ Fe mineralization throughout the oxidized and acidified soil profile, which was the opposite to pyrite and illite. This result provides powerful evidence for the preferential existence, reactivity, and redox processes of Sch, including the transformation of Sch or 2-line ferrihydrite^48^ to crystallin Fe oxyhydroxides.

Besides direct characterization, these technologies are now used to monitor As and Cr ions' ads/desorption on the Sch surface. In 1996, synthetic Sch, where the sulfate had been substituted by As and Se, was examined by X-ray absorption structure to characterize the location of anionic complexes. The results showed the direct substitution of SO_4_^2−^ by Se within tunnels and the sorption of Se outside the tunnels. However, As was mainly found outside the structure, which destabilized it and reduced growth.^49^ The X-ray absorption fine structure (XAFS) results for As adsorption on Sch^50^ showed that As was bound to Fe octahedral, and individual Fe octahedral in the Sch became more regular after adsorption.

#### Pore structure

Generally speaking, Sch is a mesoporous material. The SSA values for Sch reported to date are in the range of 2–330 m^2^ g^−1^. This suggests that more extreme production methods lead to higher SSA values. Usually, the SSA value increases as the structure becomes more complex. However, because of its poor crystallin structure, the SSA value of Sch does not strictly follow the usual fabrication methods and condition rules. As Table 3 shows, only a few reports have directly described the pore structure of Sch as mainly having a “bimodal distribution”^51^ with micropores (nearly microscale) and mesopores (mesoscale).^37,52^ There is very little mention of macro-pores. The pore size distributions (PSDs) for the micro and mesopores are similar due to the interspaces among the nanoscale villi and the aggregated Sch particles, respectively.

**Table 3:** Pore size distributions of schwertmannite samples

Source	SSA [m^2^ g^−1^]	PSDs model	*P*_micro_	*P*_meso_	Ref.
Bio. ferrooxidation	92.92	Bimodal	3 nm	30 nm	51
Fast hydrolysis	35	—	—	—	52
—	5.6	—	—	—	37

The relationship between pore/channel structure and the SSA and pore size distribution parameters for the Sch samples is not quite consistent with the relationship between fabrication method and structure/morphology. Pore size distribution is mainly decided by the primary formation of mineral nanoparticles, whereas SSA is preferentially confirmed by the secondary stacking of primary nanoparticles during nucleation and aging. This may explain some of the structure/morphology aspects of Sch, but more research needs to be undertaken in this area. Zhang *et al.*^53^ introduced fractal geometry (*D*_f_), a novel mathematical tool, to describe the irregular and complex geometries of Sch that involves morphological characteristics and pore structures. The surface (shown by denoised SEM images) and inner core (shown by low-pressure N_2_ adsorption analysis) *D*_f_ values are considered to be the operative measurements of surface roughness and pore complexity, respectively. However, this structural evaluation model is based on microstructure and the entire morphology, which means that it needs further research and improvement.

#### Electro-kinetic (E-K) properties

Usually, the E-K properties of Sch are explored by measuring its zeta potential at various pH values. More specifically, there should be a greater focus on the surface functional groups, surface charge density, zeta potential, and isoelectric point (pI) if more information about the interface between Sch and the environmental system is to be obtained.

Due to the structural similarities between Sch and ferrihydrite, it is easy to deduce that the most common functional group on the surface of Sch is Fe–OH. The Fe–OH bands exist widely in Fe-based clay or minerals.^54^ The surface charge density of a Sch powder sample can be calculated *via* the experimental method reported by Yang *et al.*^55^ or simulated by a theoretical model.^56^ The surface electric properties are key factors and are based on the Zeta potential.^57^ Two of them are mentioned frequently. These are the pH value at zero point of charge (pH_zpc_) and the pI value, which are the pH values of materials or systems^58^ when the surface charge becomes zero and the surface charge is not affected by a solution (not necessarily zero). The pH_zpc_ values of Sch samples are reasonable and varies from 3.0–7.2 depending on the fabrication and test methods/conditions. The pI value order (called pH_zpc_ in most reported references) is as follows: 7.2 (AMD)^45^ > 5.4 (bio-ferrooxidation)^59^ > 5.1 (chem. oxidation)^34^> 4.2 (fast hydrolysis)^60^ > 3.7 (fast hydrolysis)^61^ > 3.05 (fast hydrolysis).^62^ According to the above order, natural Sch may have a higher pH_zpc_ value, and Sch obtained using fast hydrolysis may have the lowest pH_zpc_ value.

#### Other properties

The sample colour is the direct impression of the final product. On most occasions, it's the easiest way to judge whether the production process has been effective or not. However, the colour is dependent on many factors, such as the drying temperature and the water percentage. Generally, the surface colour of Sch powders can be red, orange, yellow, ochre, brown,^53^ or even black, which may be due to the Fe sulfate. Previous experiments have shown that colour is closely connected with the synthetic methods, drying methods, and crumbling methods used.

The estimated log *K* values for Sch are 2.01 ± 0.30, which seems to vary depending on the sulfate content. In comparison, the value for 2-line ferrihydrite is 8.46 ± 1.40 and 10.12 ± 0.74 for 6-line ferrihydrite.^1^

### 2.3 Stability

Although Sch has a tunable chemical composition and a unique pore/channel structure, its practical application is still somewhat limited. There are two possible reasons: unstable fabrication and storage, both of which originate from the low crystallinity of the Sch phase. For example, Sch can reductively transform into two 2^nd^ Fe^2+^ minerals: siderite and mackinawite, and also relatively rapidly into goethite in *ca.* 25–65% anoxic reducing sediments,^63,64^ depending on the SO_4_^2−^ concentration.^37,65^ However, many factors can influence this transformation process, such as pH, Fe^3+^ concentration, thermal treatment, existence of organic matter, and coexisting ions, *etc.*

#### Environmental pH

Lake water samples almost all fall on a straight line for the relationship between Sch, pH value, and Fe^3+^ concentration. This reflects the solubility of Sch and shows that there is an equilibrium between them. Regenspurg *et al.*^20^ discovered that the transformation rate of synthetic Sch into goethite rose with increasing pH between pH 2–7 after a 1 year experiment. This suggested that Sch is the first mineral formed after oxidation and hydrolysis of a slightly acidic (pH 5–6) Fe^2+^–SO_4_ solution, a process that directly affects the pH of the receiving water. The transformation of synthetic/natural Sch to goethite was studied in water with variable pH (2, 4, 6 and 8) for 1 year.^66^ Only synthetic and one natural sample transformed to goethite with decreased oxalate solubility of Fe and trace elements and increased total Fe/S ratios and SSA. At pH 2, synthetic Sch fullytransformed to goethite, but at pH 4 and 6 only minor transformation occurred.

#### Concentrations of Fe species

Depending on the stability of pure Sch under Fe^2+^_aq_-rich anoxic conditions,^24^ Fe^2+^ sorption onto Sch is pH dependent, and surface coverage with Fe^2+^ appears to be the key factor controlling transformation. Furthermore, a brown precipitate of ferrihydrite was always formed after the hydrolysis of Fe^3+^ at OH/Fe^3+^ ratios >3.3. However, lower ratios induced the occurrence of ochre Sch.^52^ The effect of dissolved Fe^2+^ (0–1.0 mM) on the stability of Sch (∼40 wt% Fe^3+^ and 15 wt% SO_4_^2−^) in anoxic alkaline conditions (pH = 8) was investigated by Paikaray *et al.*,^67^ who found that product formation was accelerated when Fe^2+^ levels increased. However, the sorbed AsO_3_^3−^ markedly hindered lepidocrocite formation and almost 50–57% of the SO_4_^2−^ was released in the absence of Fe^2+^. Therefore, the correct concentrations of Fe species are strictly required for the stable existence of Sch.

#### Temperature

Thermal transformation is another method that can be used to change Sch texture. Sch transforms into jarosite, hematite, and basic Fe sulfate at relatively low temperatures.^5^ Basically, the crystal phase of Fe sulfate begins to form during thermal treatment, which triggers a series of domino effects. The thermal treatment discussed in this review includes two different paths, which are heating at low temperature during fabrication and calcination at high temperature after fabrication.

French *et al.*^43^ used high resolution transmission electron microscopy to show that the synthetic temperature in the chemical oxidation process used to produce Sch with “pin-cushion”-like morphology affects the density of needles on the surface. The needles on Sch produced at higher temperatures have a dendritic morphology, whereas those on Sch produced at room temperature are more closely packed. The low temperature treatment may cause different structural changes compared to the high temperature treatment. This factor does not change the inherent structure but does affect the acquired properties. Qiao *et al.*^44^ reported that the temperatures used during the bio-Sch process do not greatly change Sch up to 250 °C. Above this, there is a broad peak at 2*θ* ≈ 30–35°, which indicates a change from Sch to 2-line ferrihydrite (especially above 350 °C). The SSA and As removal increased when the sample was heated at 250 °C. However, removal then decreased at 550 °C, but this was dependent on the loss of –OH from the Sch surface. Johnston *et al.*^68^ used higher thermal temperatures (200–800 °C) to transform As^5+^–Sch. Their results suggested that Sch was transformed into a nanocrystalline hematite with a greater SSA and a smaller particle size when heated to temperatures above 400 °C.

#### Organic matter

Potential Fe^3+^ coordination with chelating ligands, such as –COOH and –NH_3_, may facilitate the solubilization of Fe-bearing minerals, which would significantly affect their bio-geochemical cycling.^69,70^ Organic matter, such as amino acids, is capable of inducing redox reactions and involves various sorption mechanisms.^71,72^ A typical 4-step mechanism was proposed by Xie *et al.*^73^ for the interaction between l-tryptophan and CrO_4_^2^–Sch, in which l-tryptophan acts as an inductor that causes the dissolution of CrO_4_^2−^–Sch. This involves mass transfer (adsorption of l-tryptophan onto the Sch surface) and charge transfer (reduction of Cr^6+^ to Cr^3+^). The presence of various functional groups, such as –OH, –COOH, and –OH, means that organic matter is capable of carrying out different functions, such as redox reactions and binding processes.^74^ Therefore, it strongly interacts with mineral oxides (*e.g.*, Sch, ferrihydrite, goethite, and hematite) and contaminants, such as As.^75^ There are generally two processes by which organic matter affects the transformation of Sch. These are reduction and adsorption, and, to date, adsorption has been more extensively reported than reduction.

Fulvic acid (FA) and humic acid (HA) are also often present when Sch is applied.^76,77^ Fulvic acid is well-known to interact strongly with both Sch and As. Furthermore, As concentration has a strong influence on the liberation of acidity from pure Sch and As–Sch.^78^ However, knowledge about the fate of Fe and Cr after the reduction and complexation of CrO_4_^2−^-substituted Sch by FA is poorly understood. The interaction between FA and CrO_4_^2−^–Sch was investigated by Xie *et al.*,^79^ who showed that the Fe and Cr concentrations in solution increased at first, but then decreased when FA was at 10 mg L^−1^ and the pH was 6.5. This was possibly due to synergistic effects, such as interactions between ligand-promotion and reduction. The effect of FA on the liberation of acidity and the mobilization of As results in a greater liberation of acidity by As–Sch at pH 4.5 and 6.5 compared to pure-Sch.^78^ The FA enhanced As mobilization at both pH values, but it was greater at pH 6.5. Humic acid has a skeleton of alkyl and aromatic units with carboxylic acid, phenolic hydroxyl, and quinine functional groups.^80^ These large molecules can strongly adsorb to the outer particle surfaces (not the interior surfaces) of Sch powders. Therefore, these organic molecules can effectively modify species mobilization and acidity liberation. Furthermore, FA seems to affect Sch transformation more than HA.

#### Coexisting ions

Pure solutions with only one solute usually do not exist in natural water or soil environments. This means that the influence of other cations and anions needs to be considered. Acero *et al.*^81^ used Sch to remove As from a solution with divalent trace metals (Zn, Cu, Pb, Cd, Ni, and Co) and found that the concentration of metal cations remained almost unchanged. When Dou *et al.*^82^ used granular Sch to remove As, they found that Cl^−^, SO_4_^2−^, NO_3_^−^, and CO_3_^2−^ did not inhibit the adsorption of As oxyanions, whereas coexisting PO_4_^3−^, SiO_4_^4−^, and HA caused increased interference as their concentrations rose in the order HA > PO_4_^3−^ > SiO_4_^4−^. Research has shown that organic coexisting ions and P-based ions clearly influence the removal of As by Sch due to the strong interaction between organic matter and As, and because organic coexisting ions and P-based ions have similar chemical properties. With regards to CrO_4_^2−^, the competition by the accompanying anions with CrO_4_^2−^ for adsorption sites followed the order H_2_PO_4_^−^ > SO_4_^2−^ > NO_3_^−^.^83^ Gan *et al.*^84^ reported that Cu^2+^ produces a remarkable decrease in Cr removal and a quite similar order of H_2_PO_4_^−^ > SO_4_^2−^ > NO_3_^−^ > Cl^−^ for the single removal of CrO_4_^2−^. In conclusion, organic coexisting ions have a greater influence on Sch adsorption ability than inorganic ones and, more specifically, the metal cation and oxyanion effects on the adsorption process are strongly dependent on the pollutant species.

The factors mentioned above not only affect the formation of “fresh” Sch, but also influence the long-term storage of Sch products. When Sch is formed in AMD, its transformation to goethite occurs within 2 years in meteoric waters.^85^ Regenspurg *et al.*^20^ suggested that Sch occurrence is transient and restricted to certain environments, such as acidic mining lakes, where Fe^3+^ coordination is controlled by competition between SO_4_^2−^ and OH. In contrast, synthetic Sch samples are more chemically stable and can remain unchanged after years. This is due to their higher purity and better storage conditions compared to natural Sch forms, as previously mentioned. Furthermore, pH is an important factor that affects the long-term chemical stability of Sch. The effect of pH on the release of As from Sch–As is more obvious at higher pH values. This indicates that As release from Sch–As is mainly controlled by outside environmental factors, such as pH, rather than time.^86^

## 3. Environmental remediation application

### 3.1 Water treatment

Over recent decades, there have been many systematic studies on Sch and its potential use in environmental remediation.^87^ They have mainly focused on the removal of contaminants in the water phase, such as AsO_4_^3−^, CrO_4_^2−^, SbO_4_^3−^, and F^−^*etc.* These reports provide a solid foundation for theoretical research. The details are listed in Table 4.

**Table 4:** Composition, structure and potential applications of schwertmannite

Source	Composition		Structural/E-K parameters			Conditions						Adsorptions	Ref.
	Fe/S	SO_4_^2−^ %	Morphology	*S*_BET_ [m^2^ g^−1^]	pH_zpc_	Pollutant^a^	*C*_0_ [mM]	*S*/*L* [g L^−1^]	pH^b^	*T* [°C]	*T* [h]	*Q*_e_ [mmol g^−1^] or *Q*_m_ [mg g^−1^]	
AMD or AMLs	1.3–16	32–19.4	Large spheroids	72 (ml 77)	—	—	—	—	—	—	—	—	20
	5.37	16	—	173	2	—	—	—	—	—	—	—	45
	4.35	15.0	Sphericity	80 ± 8	—	As	Trace	—	—	—	—	—	81
	4.60	—	Spherical/elliptical	—	—	—	—	—	—	—	—	—	15
	5.30	—	Spheroidal	14.7	—	As^III^	0.067–1.33	5–100	—	2–25	2	*Q*_e_: 0.0126	104
Chemical oxidation	3.81	—	—	—	—	—	—	—	—	—	—	—	20
	5	—	—	4–14	—	As^III^	0–60	68.0	9.0	—	60	*Q*_m_: 280.0	32
						As^V^			3.0			*Q*_m_: 246.0	
	4.71	16.3	Smooth rounded	5.3	—	As^III^	0.13–1.33	10	2.6	—	120	*Q*_m_: 14.16	38
	—	—	40 nm	—	—	—	—	—	—	—	—	—	35
	4.32	16.2	Spheroids	—	—	—	—	—	—	—	—	—	78
	—	—	Closely packed	—	—	—	—	—	—	—	—	—	43
	—	—	Dendritic	—	—	—	—	—	—	—	—	—	43
	—	—	Aggregates	130.9	7.1	Sb^V^	0–0.49	0.3	7.0 ± 0.2	5 ± 1	24	*Q*_m_: *ca.* 90	34
	4.75	—	Spheroidal	—	—	—	—	—	—	—	—	—	63
	4.71	—	Spheroidal	10	—	—	—	—	—	—	—	—	64
	4.17	—	Sphere shape	48.18	—	—	—	—	—	—	—	—	89
	4.67–5.04	—	Spheroids, hedge-hog	2.06–16.30	—	As^III^	(3.33–13.3) × 10^−4^	0.25	7.0	30	24	—	31
	5.30	—	Spheroidal	50.4	—	Nitro-Ph	—	—	—	—	—	—	33
Fast hydrolysis	4.04–4.82	15.1–16.1	—	—	—	—	—	—	—	—	—	—	1
	—	—	—	165	4.2	Cr^VI^	—	—	4.2	—	5	*Q*_m_: 178.6 mg g^−1^	60
	4.57	—	Hedge-hog like	325.52	3.05	—	—	—	—	—	—	—	62
	—	—	—	206.1	—	As^V^	(10–35)	0.3	5.0	5 ± 1	24	*Q*_m_: (104.2)	82
	5.30	5.31	—	—	—	As^V^	—	1.0	3–4	5	24	*Q*_m_: 114	6
	—	—	Hedge like	—	3.7	As^III^	(0.2–0.6)	0.25–2	3–11	25	4	*Q*_m_: 1.011	61
	4.57	—	Hedgehog-like	325.52	—	AsV	1.33	1	3	5	5	Qm: 182.86	89
						AsIII			7	25		Qm: 217.85	
	—	24 ± 1	Agglomeration	5.6 ± 1.2	—	—	—	—	—	—	—	—	52
	—	—	—	199.43	—	SbII	0.181	0.5/1/2/4	7.0 ± 0.2	25 ± 1	48	Qm: 84.03	92
						SbV	0.189					Qm: 78.60	
Slow dialysis	—	13.5	—	154	—	AsV	0–100.5	1	3.95 ± 0.04	5	24	Qm: 82.4	26
	6.15	—	Hedge-hog	—	—	—	—	—	—	—	—	—	20
	—	12.3	—	—	—	—	—	—	—	—	—	—	45
	—	—	—	—	—	—	—	—	—	—	—	—	46
	5.30	15.4	Uneven surfaces	210.0	—	AsIII	0.13–1.33	10	2.6	—	120	Qm: 20.08	38
	7.84	9.79	—	171	—	As	0.5–4	1	4.5–4.6	25 ± 1	72	Qm: 196 mmol/molFe	102
						Cr	0.15–2		4.5			Qm: 115 mmol/molFe	
						Mo	0.2–2.5		4.5			Qm: 88 mmol/molFe	
	—	—	Aggregate	—	—	—	—	—	—	—	—	—	35
	—	—	Irregular-shaped	199.43	—	AsV	0.02–0.27	0.5	7.0	25 ± 1	24	Qm: 31.7	82
			Cylindrical-shaped	189.27								Qm: 22.3	
			Spherical-shaped	32.52								Qm: 3.6	
	5.13	—	—	—	—	HAsO42-	0–20	1	7.00 ± 0.28	25	24	Qm: 76.6	103
						CrO42-						Qm: 37.6	
						SeO42-						Qm: 24.7	
	—	—	Macropore/network	14.81	—	HA	—	—	—	—	—	Qe: 15.1	53
						CrVI						Qe: 4.2	
Biological ferrooxidation	6.4	12.5	Fibrous aggregates	14.7	—	AsIII	0.13–1.33	10	3.1	—	120	Qm: 103.39	38
	4.7	14.9	Spheroidal	14.7	—	As^III^	2.0	10	3.0	25	132	*Q*_e_: 3.746	90
	—	—	—	—	—	Cr^IV^	10	0.1	2–8	—	—	*Q*_m_: 35.30	83
	—	—	Spherical, net like	—	—	Cu^II^	0.31–3.15	—	8.0	—	24	*Q*_m_: 10.35–50.14	84
						Cr^VI^	0.39–3.85		5.0			*Q*_m_: 30.46–38.82	
	—	—	Needle, whiskers	—	—	—	—	—	—	—	—	—	40
	5.16	—	Net-work like	33.52	—	As	—	—	—	—	—	—	41
	—	—	—	—	—	As	—	—	—	—	—	—	42
	—	—	Spherical	5.14	—	As^III^	0.013	0.25	7.50	28	4	*Q*_e_: 1.004	44
	—	—		110.06–78.82	—							*Q*_e_: 3.72–2.928	44
	—	—	Sea urchin, flower	34.55	—	HA	—	—	—	—	—	*Q*_e_: 12.3	53
						Cr^VI^						*Q*_e_: 5.5	
	6.3	—	—	14.7		—	—	—	—	—	—	—	24
	4.65	—	Spheric	92.92	—	BPA	—	—	—	—	—	—	51

a The relative atomic masses of As (74.92 ≈ 75), Cr (51.996 ≈ 52), Cu (63.546 ≈ 64), Sb (121.75 ≈ 122), and Se (78.96 ≈ 79), respectively.

b The optional pH for adsorption is chosen as a defined value rather than a value range, if provided.

#### Removal of pollutants

So far, the pollutants that can be removed or stabilized include metal pollutants like AsO_4_^3−^/AsO_3_^3−^, CrO_4_^2−^, SbO_4_^3−^/SbO_3_^3−^, Cd^2+^, Cu^2+^, *etc.* and other inorganic pollutants like fluorions. Also, Sch can be applied as effective catalyst for organic degradation. Here, this paper will discuss the remediation ability of Sch from these two aspects.

#### Metals and other inorganics

##### AsO_4_^3−^ and AsO_3_^3−^

The presence of As in water and soil systems (dominantly originating from parent rock and human activities) is a serious environmental problem for humans and other living organisms due to its high toxicity and wide distribution in natural environment.^88^ Even at low concentrations, long-term exposure to inorganic As such as AsO_4_^3−^ or AsO_3_^3−^ can lead to several diseases. Usually, the valence state of As needs to be considered, and as the concentration and pH should be taken into account when predicting and managing As mobility in Sch rich systems.

In general, sorption of AsO_4_^3−^ is greatest at low pH, whereas high pH favours the sorption of AsO_3_^3−^. Burton^32^ showed that the actual pH of equivalent AsO_4_^3−^ and AsO_3_^3−^ sorption was strongly load dependent when the pH decreased from ∼8.0 to pH ∼4.6. AsO_4_^3−^ is strongly partitioned to the Sch solid phase at low loadings while AsO_3_^3−^ is weakly adsorbed at low loadings, but has a greater affinity at high loadings. The sorption of AsO_4_^3−^ and AsO_3_^3−^ causes a significant release in SO_4_^2−^ from within the Sch solid-phase, which can be interpreted as As sorption *via* incorporation into the Sch structure rather than merely surface complexation at the mineral–water interface. Similar results were also reported by Song *et al.*,^89^ who found that more AsO_4_^3−^ was adsorbed on Sch at lower pH values, whereas AsO_3_^3−^ sorption increased as the pH rose.

The As loading may also have different influences on its removal. Sorption is governed by multilayer processes, as indicated by highly nonlinear Freundlich adsorption isotherms.^38^ A relationship between AsO_3_^3−^ uptake and sulfate release is only observed at high initial AsO_3_^3−^ concentrations, which suggests that AsO_3_^3−^ retention through ligand exchange is of minor relevance. Higher AsO_3_^3−^ loadings cause morphological degradation, which lead to angular particles with porous centres, and the extent of the degradation is affected by AsO_3_^3−^ partitioning. Biogenic Sch with an SSA of 14.7 m^2^ g^−1^ was reported by Paikaray *et al.*^90^ who showed that there was a rapid AsO_3_^3−^ uptake followed by slow retention, possibly into the internal absorbing sites through multilayer and heterogeneous sorption processes. The ionic exchange between SO_4_^2−^ and AsO_3_^3−^, and surface precipitation governs the total AsO_3_^3−^ uptake, where lower dissolved SO_4_^2−^ and a higher sorbent mass enhance AsO_3_^3−^ retention. The AsO_3_^3−^ ratio affects surface oxidation to AsO_4_^3−^, whereas AsO_3_^3−^ is the predominant redox species at high AsO_3_^3−^ : Fe^3+^ ratios. Only 0.83% of adsorbed AsO_3_^3−^ was released, which was subsequently re-adsorbed into Sch during a 4 months stabilization period without mineralogical transformation.

Schwertmannite can also be produced in other forms or shapes, such as irregular, cylindrical, and spherical granules.^82^ The irregular and cylindrical granules have larger Sch loadings, higher porosities, a more abundant pore structure and larger *V*_micro_ values than the spherical forms. The AsO_4_^3−^ adsorption kinetics follows a pseudo second order rate equation and there is a two-stage of intraparticle diffusion process. The diffusion rate parameter order is irregular granules > cylindrical granules > spherical granules, which is in accordance with their *V*_p_ and interparticle porosity. Furthermore, *Q*_e_ values of 34, 21, and 5 mg g^−1^, for irregular, cylindrical, and spherical granules, respectively, are achieved at an initial AsO_4_^3−^ concentration of 20 mg L^−1^ and 0.5 g L^−1^. Irregular and cylindrical granules perform much better over a wider pH range than spherical granules and can be used for four cycles without reducing the *Q*_m_ value. Furthermore, the toxicity characteristic leaching procedure (TCLP) results show that the spent irregular and cylindrical granules are inert and could safely be disposed of in landfills.

##### CrO_4_^2−^

Chromium is a heavy metal contaminant that is frequently detected in the natural environment. It poses a threat to fauna when present as the CrO_4_^2−^ species due to its higher solubility, mobility, and toxicity, when compared with Cr^3+^.^91^ Previous studies have shown that Sch used for CrO_4_^2−^ removal is usually prepared by biological ferrooxidation of the Fe^2+^ species. Many batches are prepared using slow hydrolysis.

Biogenic Sch has been used to absorb CrO_4_^2−^ in a continuous flow column (1 mL min^−1^, pH = 6) with a *Q*_m_ value of 35.30 mg g^−1^. A possible adsorption mechanism for CrO_4_^2−^ is the anion exchange process between various Cr^6+^ species and SO_4_^2−^, a constituent of Sch.^83^ Gan *et al.*^84^ modified biosynthetic Sch with AlPO_4_. The results showed that the bio-Sch changed from villous spherical aggregates to smooth globules. Furthermore, crystallinity decreased as the AlPO_4_ content increased. The optimum pH for CrO_4_^2−^ adsorption was at 5.0. The *Q*_*m*_ for CrO_4_^2−^ reached 38.82 mg g^−1^. The modification enhanced CrO_4_^2−^ selective adsorption in a binary metals system. Furthermore, the materials could be effectively regenerated after they were washed in pH 2.0 water. Overall, the biogenic Sch samples had limited adsorption CrO_4_^2−^ capacities, even under acidic conditions, which favour CrO_4_^2−^ remediation. Therefore, the CrO_4_^2−^ absorption properties of Sch produced by alternative methods need to be discussed. Previous studies have suggested that Sch with relatively ordered channel structures may be more effective at CrO_4_^2−^ removal. Therefore, the preparation methods and conditions could possibly be a key issue that needs to be addressed by future research.

##### SbO_4_^3−^ and SbO_3_^3−^

Compared to As and Cr, the references about Sb removal by Sch are easily summarized and much fewer in number. In general, the studies on Sb removal by Sch are quite similar to those about As, but are still limited to water phase removal. Previous research suggests that Sb is not easy to remove from water, even though it is in the same family as P and As, Dong *et al.*^34^ combined Sch in its main active phase (confirmed by X-ray photoelectron spectroscopy) with graphite oxide (GO) as a composite adsorbent for SbO_4_^3−^ removal so that there could be a synergistic effect between Sch and GO. The *Q*_m_ value for SbO_4_^3−^ was 158.6 mg g^−1^ (8.0 mg L^−1^ SbO_4_^3−^, pH = 7.0), which was superior to either GO or Sch alone. This was due to the effective incorporation of Sch among GO platelets and its high dispersion on the GO surface. They also used Sch granules to overcome the drawbacks of using small particle-sized adsorbents for SbO_3_^3−^ and SbO_4_^3−^ removal from water.^92^ Sch granules were able to remove 32.9 mg g^−1^ SbO_3_^3−^ and 23.2 mg g^−1^ SbO_4_^3^, which was superior to most reported granular and powdered absorbents. The SbO_3_^3−^ was effectively removed over a wide pH range, while the removal of SbO_4_^3−^ was pH dependent and could be enhanced by lowering the solution pH. The SbO_3_^3−^-loaded Sch was regenerated with 91.2% re-adsorption capacity, and this could be repeated five times. However, the Sch adsorption capacity for Sb still needs further research because it is toxic even at low concentrations in water.

##### Fluorions

Fluorions have a small volume, which means that they easily enter into channel pores to form stable complex compounds. Related studies have shown that many Fe oxides or hydroxides have good adsorption capacities for fluorions, especially, when the pH value is lower than the pH_zpc_ value for Sch. In addition, Sch also possesses many –OH and SO_4_^2−^ groups on the surface, which also exchange with fluorions. Methods involving Sch in the water phase and within the soil system have great potential compared to traditional methods for fluorion removal.

Research by Eskandarpour *et al.*^93^ into the adsorption of fluorions onto Sch in a batch system suggests that the adsorption of F by Sch is high, but it is insensitive to changes in temperature and equilibrium pH. The inner-sphere complex-forming species had negative effects on fluorion adsorption, whereas outer-sphere complex-forming species slightly improved fluorion removal efficiency. A nano magnetic Sch prepared by introducing nano-magnetite into Sch^94^ achieved the permissible limit defined by World Health Organization for removing F from water with a *Q*_e_ of 17.24 mg g^−1^ according to the isotherm data. Interference by PO_4_^3−^, CO_3_^2−^, and HCO_3_^−^ was more than by Cl^−^ and SO_4_^2−^. The spent adsorbents were regenerated with basic solutions and they retained good adsorption efficiencies.

##### Cd^2+^, Cu^2+^, *etc*

Schwertmannite can also remove cationic contaminants. For example, Cd is a typical toxic contaminant and needs to be removed from wastewater. Fan *et al.*^95^ investigated the feasibility of using slow synthetic Sch to remove Cd^2+^ from aqueous solutions. They used batch experiments that tested different Cd^2+^ concentrations, temperatures, and pH values. The Langmuir isotherm successfully described the adsorption of Cd^2+^. A thermodynamic study suggested that absorption was spontaneous and endothermic. The Sch had a high Cd^2+^ adsorption capacity of 110 mg g^−1^ at a dosage of 1 g L^−1^ and an initial pH of 8.0 at 25 °C. At pH > 6.0, Cd adsorption dramatically increased and nearly 100% of the Cd was adsorbed at pH 8.0. In the natural pH range for Sch, the Sch that had adsorbed Cd^2+^ had good regeneration ability. The Cd^2+^ desorption proportion of the total sorbed quantity was 50–80% and the desorption rate increased as the pH decreased. Therefore, Sch can be employed as an efficient adsorbent for the removal of Cd from contaminated water. However, there are many other heavy metals, such as copper,^84^ that could be removed by Sch or Sch-based materials, but these are not discussed in this review.

#### Organic

Schwertmannite also plays an important role as a catalyst in the degradation of organic pollutants. Organic pollutants that can be removed by Sch are mainly phenyl compounds, such as phenols. For the first time, Wang *et al.*^62^ applied Sch as a Fenton-like catalyst in the oxidation of phenols by H_2_O_2_ and experimentally confirmed its good catalytic activity, especially in high salinity systems. Sch was more effective than goethite and could maintain phenol removal at 98%, even after 12 cycles, although a phase transformation of Sch to goethite was observed. Duan *et al.*^33^ used Sch in another Fenton-like process for nitrobenzene degradation. In this case, its catalytic activity was maintained at 92.5% (30 min, pH 3.0, 500 mg L^−1^ H_2_O_2_) after five runs. Yang *et al.*^96^ developed an *in situ* remediation technology that coupled the Sch/H_2_O_2_ process with the E-K process for the removal of phthalates and acetaminophen in river sediments and found its remediation performance was superior to that of the batch degradation test. Clearly, Sch is a promising Fenton-like catalyst and its performance can be further enhanced by combining it with other technologies. Recently, Li *et al.*^51^ established a Sch/H_2_O_2_ system that also used ultrasonic technology for bisphenol A degradation. Removal was significantly enhanced to 98% compared to the Sch/H_2_O_2_ system at 69.6%. Furthermore, this remained at 95% after five cycles. Although there have only been a few application examples for organic remediation by Sch to date, it is clear that Sch can be combined with a number of traditional catalytic process, which means that it will play a more important role in the future.

As we concluded in Table 4, among all the related pollutants, Sch presents better adsorption properties for As and Cr-based pollutants than the other types. This is mainly caused by the unique channel-like structure whose pore diameter is quite close to the size of As or Cr-based ions like AsO_4_^3−^, AsO_3_^3−^, or CrO_4_^2−^, promoting the easy ion-exchange process. As for As-based pollutants, the Fe element in Sch phase can be also used to firmly bond with As-based ions to form more stable phase. This point can also be confirmed by the amounts of literatures, as a proof.

#### Regeneration in water systems

With regards to “green chemistry,” Sch, especially the synthetic versions, are expected to be utilized in regeneration and recycling processes in the future. Typically, for As remediation, recovery by NaOH solution (0.1–1.0 M),^44,82,92,94^ washing with deionized water (>5 times),^51^ and dry processing (40–60 °C) are common processes that ensure that the adsorbates can be nearly completely separated without the Sch structure being destroyed. It is worth noting that under the alkaline condition, Sch can transform into other phase possibly, which is not discussed too much before. As for Cr^VI^ and Cu^II^ remediation, a diluted H_2_SO_4_ solution (pH 2.0) was used for regeneration of Sch considering that Cr^VI^ can be desorbed from active sites through washing with low pH deionized water.^84^ It turns out to be that there is no appreciable decrease in both metals owing to the well-kept morphology. Compared to the adsorption process, Sch used as catalysts for organic degradation are much easier to regenerate only by washing with deionized water several times and dry.^51^

These processes are not time/energy-consuming, which means they may become common Sch regeneration techniques. After regeneration, the adsorption capacity of Sch decreases to a certain degree, but it can still reach the adsorption capacity of fresh batches if the adsorption conditions are adjusted. There are numbers of reasons why Sch can be easily regenerated. It is possible that the basic aqueous solution destroys the surface bonds on the outer surface of Sch or the inner surface of the Sch channels. Fast washing and quick separation without complex processes and special reagents do not influence the Sch structure, which ensures that the adsorption capacity of Sch remains high in subsequent cycles. This easy separation of solid Sch and the regeneration process gives this material wide applications as adsorbent or catalyst in heterogeneous systems. Sch could also be reobtained by using some other functional composites, such as magnetic nanoparticles or hydrophilic/hydrophobic molecules. However, these possibilities require further research.

### 3.2 Soil remediation

Considering the huge amount of researches about Sch for water treatment, the potential of Sch for treatment on inorganic and organic pollutants could also be technically applied in soil remediation. The first research on the use of Sch for soil remediation appeared in 2016. However, there have not been many studies to date. Biogenetic Sch pre-treated by acidic and alkaline activation (A-Sch)^41^ achieved an immobilization efficiency of > 99.5% for water-soluble As with an A-Sch dosage of 5% and an Sch dosage of 10%, respectively, in As-contaminated soil. The immobilization percentages for NaHCO_3_-extractable As increased from 31.5% to 90.4% and from 40.2% to 93.8% when the dosage increase from 0.5 to 10 wt% for Sch and A-Sch, respectively. In general, both Sch and A-Sch effectively immobilize As in contaminated soil, and the immobilization performance of A-Sch is better than Sch, especially at lower dosages. This finding also has important implications for *in situ* immobilization of As in contaminated soils, especially in soils that have acidic Fe and sulfate-rich environments. This study was finished in 2016 and used an indirect application of Sch. The combination of microbial oxidation (AsO_3_^3−^-oxidizing bacterium strain YZ-1) (CGMCC No. 8329) and bio-Sch immobilization by Yang *et al.*^42^ successfully treated highly As-contaminated soil with immobilization efficiencies of 99.3% and 82.6% for water-soluble and NaHCO_3_-extractable total As, respectively. This study suggested that the combination could potentially remediate highly As-contaminated soils.

Studies about farmlands or other sites are needed so that the Sch remediation of heavy metal contaminated soils can be further evaluated. Sch is quite an effective remediator of As-contaminated soil and perhaps Sb and Cr contaminated soil, although there have been no published reports on these latter two metals. Furthermore, potential problems about production technology, quality control, and cost control have still to be resolved, and the sustainability of contaminated soil remediation by Sch also needs to be examined.

As comparisons, Schs especially the synthetic ones own a comprehensive advantage for water and soil remediation. For example, Sch shows much higher SSA value than the reported organo-bentonites,^97^ as well as wide application for more types of heavy metals. This mineral also needs facile fabrication, compared to the reported magnesium silicate-hydrothermal carbon composite^98^ and graphene oxide (or its composites).^99,100^ As for the adsorption for ionic typed pollutant like As^III^, Sch shows a significant advantage with much higher adsorption than the reported CaMgFe-LDH.^101^ Technically, the Sch mineral is an easily prepared, more effective, and more potential candidate for types of environmental process, even as a catalyst or carrier.

## 4. Remediation mechanisms

### 4.1 Inorganics

**Fig. 6:**
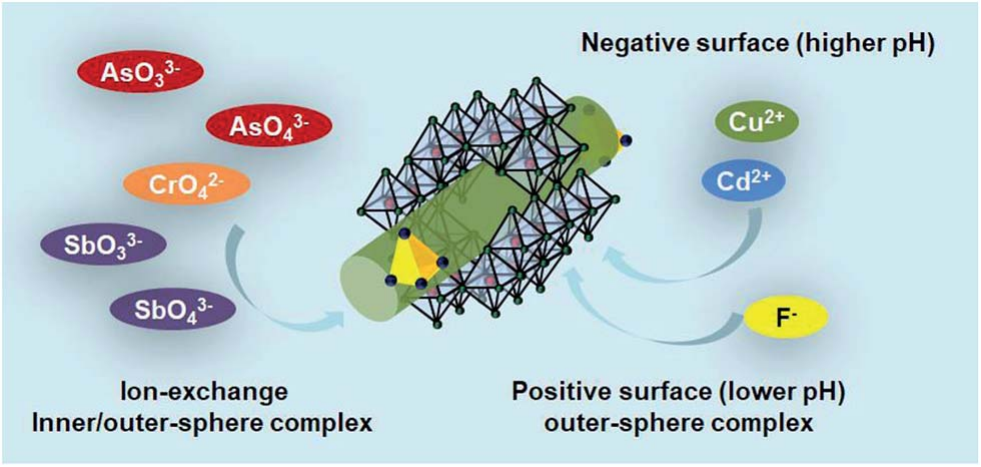
The treatment mechanism of As, Sb, Cr, Cd, Cu, and F-based pollutants by schwertmannite in water.

It is unrealistic to propose only one Sch method for the removal of heavy metals. Antelo *et al.*^102^ proposed two mechanisms: complexation with Fe hydroxyl surface groups and anion exchange with sulfate complexes. Arsenic oxyanion adsorption mainly occurs through the 1^st^ mechanism, whereas CrO_4_^2−^ oxyanions are absorbed by the 2^nd^ mechanism. The adsorption and post adsorption behaviour of Sch towards various oxyanions were investigated by Khamphila *et al.*^103^ The results implied that the AsO_4_^3−^, PO_4_^3−^, and CrO_4_^2−^ Sch adsorption mechanism was different from SeO_4_^2−^ and SO_4_^2−^. The AsO_4_^3−^, PO_4_^3−^, and CrO_4_^2−^ form inner-sphere complexes, whereas SeO_4_^2−^ and SO_4_^2−^ ions form outer-sphere complexes with the Sch surface. An illustration about the main mechanism path of Sch for water treatment is presented as Fig. 6 shows.

#### AsO_3_^3−^ remediation

There are usually two paths chosen for the removal of AsO_3_^3−^. One is to directly stabilize it and the other is to oxidate it first and then chemically stabilizes it. AsO_3_^3−^ uptake by Sch is rapid and has a three-step process. In the 1^st^ step, the Sch mineral is widely believed to act as an efficient AsO_3_^3−^ scavenger in anoxic acidic environments, or it may act as an improved AsO_3_^3−^ oxidant at identical Fe^3+^/AsO_3_^3−^ ratios.^104^ In the 2^nd^ step, the Sch mineral is combined with AsO_3_^3−^ by a bidentate binuclear coordination characterized by Fe–As and As–O interatomic distances, which are almost identical to other Fe^3+^-(hydr)oxide minerals. Sorption of AsO_3_^3−^ leads to the development of AsO_3_^3−^/AsO_4_^3–^–Fe^3+^–SO_4_^2−^ or AsO_3_^3−^/AsO_4_^3–^–Fe^3+^ type surface precipitates and causes significant morphological degradation without forming new mineral phases. In most cases, Sch causes partial oxidation of AsO_3_^3−^ to AsO_4_^3−^, which detoxifies As and partly restricts As mobility in acidic anoxic environments. In the 3^rd^ step, the AsO_3_^3−^ or AsO_3_^3−^-based AsO_4_^3−^ species form a surface precipitate. Paikaray *et al.*^38^ found that an additional relatively lower absorption band appeared at ∼1388 cm^−1^ in the IR data, which was absent in As-free Sch but consistently present in all the contaminated samples and became more distinct at elevated AsO_3_^3−^ concentrations. The intensity of this peak may be directly related to solid phase AsO_3_^3−^. The appearance of this absorption band may reflect a surface precipitate of a ferric arsenite.

For AsO_3_^3−^ species, the best fitting models are the Freundlich model^38,90^ and the Langmuir model.^82^ The former one shows that AsO_3_^3−^ is retained by multilayer sorption sites with heterogeneous sorption energies^105^ and the latter model indicates monolayer sorption.

#### AsO_4_^3−^ remediation

AsO_4_^3−^ is the oxidized version of AsO_3_^3−^ and does not need an extra oxidizing process. Fukushi *et al.*^27^ concluded that the reactive sites for AsO_4_^3−^ sorption are surface coordinated SO_4_ groups rather than surface –OH groups. The AsO_4_^3−^ sorption mechanism involves ligand exchange with surface-adsorbed and structural SO_4_^2−^. AsO_4_^3−^ can be coordinated in the forms of monodentate AsO_4_^3−^ coordination at the surface-adsorbed SO_4_ sites [(Fe_1_)_2_SO_4_] and bidentate AsO_4_^3−^ coordination at the structural SO_4_ sites [(Fe_3_)_2_SO_4_]. The overall ligand–exchange reaction is as follows by formula (4):

4 0.61(Fe_1_)_2_SO_4_ + 0.39(Fe_3_)_2_SO_4_ + 1.61H_2_AsO_4_^−^→ 1.22Fe_1_H_2_AsO_4_ + 0.39(Fe_3_)_2_HAsO_4_ + 0.39H^+^ + SO_4_^2−^

#### CrO_4_^2−^ remediation

CrO_4_^2−^ has a similar ionic radius and charge to SO_4_^2−^, and tetrahedral coordinates to the surface and channels of Sch.^27^ This means that Sch has a certain selectivity for CrO_4_^2−^ and other ions with similar radii. Antelo *et al.*^102^ concluded that anion exchange mechanisms facilitate CrO_4_^2−^ removal from aqueous systems. Usually, CrO_4_^2−^ barely interacts with other iron oxides and this reduces the mobility of such ions in environments affected by AMD. It is important to emphasize that CrO_4_^2−^ ions form inner-sphere complexes with the surface of Sch.^103^ The existing form and combining sites for CrO_4_^2−^ on/in Sch structures can be examined and verified by characteristics methods, such as FI-IR, as reported by Bigham.^9^ It is easy to deduce the CrO_4_^2−^ forms and their proportional distribution according to the relative strengths of their characteristic peaks.

The best fitting models for CrO_4_^2−^ are the Freundlich^60^ and Langmuir models,^84^ which have negligible differences of *R*^2^ constant.

### 4.2 Organics

**Fig. 7:**
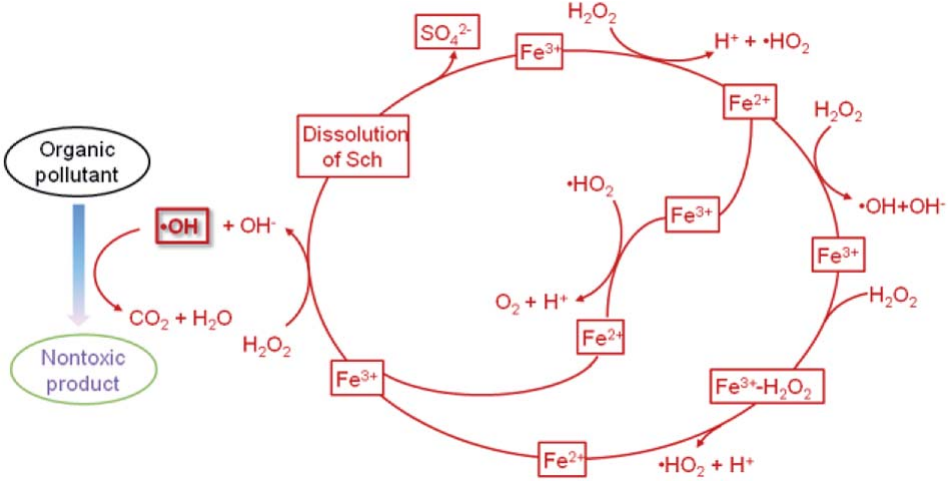
The Fenton reaction when catalysed by schwertmannite.

The treatment of organic contaminants in water or soil is best undertaken using the Fenton reaction. Although the reactants may differ, the reaction mechanism is quite similar. A possible mechanism for the oxidation of phenol with H_2_O_2_ in the presence of Sch^62^ is shown in Fig. 7. In the oxidation process, intermediates, such as quinines, catechol, oxalic acid, and acetic acid are produced. These organics play an important role in reducing Fe^3+^ to Fe^2+^ and lead to the dissolution of Fe oxides. Most of the intermediates are further oxidized and transformed into H_2_O and CO_2._

In this mechanism, the identification of ·OH is required. Methanol was chosen as an effective ·OH scavenger to identify whether the ·OH type reaction has degraded the nitrobenzene,^33^ and Fe leaching and H_2_O_2_ decomposition confirmed the success of the process. The effect of other kinds of ·OH scavengers was also investigated during the degradation of bisphenol A.^51^ In the degradation reaction, free ·OH was generated in the bulk solution along with some intermediates, including *p*-isopropenyl phenol, *p*-benzoquinone, and methyl propionate, which proved the occurrence of a Fenton-like process on the Sch surface.

## 5. Conclusions and perspectives

Schwertmannite is a potential material that can be used for environmental remediation. This mineral has a unique channel structure and ion-exchange properties, which gives it an excellent adsorption capacity for inorganic contaminants in the water phase and catalytic degradation ability for organic contaminants, resulting in potential application in environmental remediation of water and soil. In the remediation process, the stability of Sch is an important factor that significantly influences its application, owing to the easy transformation of metastable Sch into other minerals. So far, Sch is not just a mineral or clay, but also an adsorbent or catalyst. Novel functional Sch materials in further investigation are always concerned. However, there are still lots of questions about the synthesis and application of schwertmannite as follows:

(1) The schwertmannite samples fabricated *via* various methods and conditions present quite remarkable differences in material's characteristics and the adsorption properties of pollutants. The chemical oxidation, fast hydrolysis, slow dialysis, and biological ferrooxidation process are closely connected with distinct microstructures, pore-structure, and most importantly the adsorption properties. This phenomenon can be observed even at similar conditions. The relationship between the synthetic strategies or methods and materials' properties should be discussed from different views to provide more clarity about the controllable optimization aiming at the practical application.

(2) The environmental application of schwertmannite mainly focuses on water treatment while those about soil remediation are still in starting stage. Technically, the researches about the former aspect mainly revolve industrial waste water and acid mine drainage. In 2016, the study in schwertmannite for soil remediation was firstly reported with lots of attentions. Afterwards, more discussions are eagerly necessary for further application.

(3) Schwertmannite is a metastable amorphous Fe-based mineral, which is quite easy to transform into goethite phase. Such phase-transforming process has become an important and necessary issue. Schwertmannite can exist in long-term during the remediation process of soil and underground water, requiring necessity about the study for its phase-transformation and stability. Several researches about these factors of natural/synthetic or As/Cr-doped Schwertmanites at different pH, temperature, co-existing ions, and organic matter have been reported in succession. Nevertheless, the issues about how the phase transformation occurs, how the pollutants release, and how long the release will last, are still lack of related simulation and tracking study, especially in the system of underground water and soil.

(4) As a potential catalytic material, schwertmannite can be used for the degradation of organic pollutants basically as efficient Fenton-like catalysts. Until now, kinds of aromatic compounds (such as phenolic and BPA) have already been examined as candidates, and excellent catalytic property in short time has been achieved. However, its recycling ability and chemical stability during the process of chemical reactions still need more deep study.

(5) Many paths can be used for the promotion of adsorption properties of Sch to various pollutants, such as thermal modification, acidic modification, organic modification, and even shape granulations. Other strategies and technologies for structure optimization and functionalization are particularly concerned for more systematic researches.

## Conflicts of interest

There are no conflicts to declare.

## Supplementary Material
